# *LHFPL2* Serves as a Potential Biomarker for M2 Polarization of Macrophages in Renal Cell Carcinoma

**DOI:** 10.3390/ijms25126707

**Published:** 2024-06-18

**Authors:** Xiaocheng Gong, Yunfei Liu, Qian Zhang, Keying Liang, Jinfen Wei, Hongli Du

**Affiliations:** School of Biology and Biological Engineering, South China University of Technology, Guangzhou 510006, China; 202010108425@mail.scut.edu.cn (X.G.); 202120149279@mail.scut.edu.cn (Y.L.); 202110189331@mail.scut.edu.cn (Q.Z.); 201910107991@mail.scut.edu.cn (K.L.); weijinfen@scut.edu.cn (J.W.)

**Keywords:** *LHFPL2*, macrophages, M2 polarization, immune infiltration

## Abstract

Renal cell carcinoma (RCC) is one of the most common malignant tumors of the kidney, presenting significant challenges for clinical diagnosis and treatment. Macrophages play crucial roles in RCC, promoting tumor progression and warranting further investigation. Previous studies have identified *LHFPL2* as a transmembrane protein associated with reproduction, but its relationship with tumors or macrophages has not been discussed. This study utilized transcriptomic sequencing data from 609 KIRC patients in the TCGA database and single-cell sequencing data from 34,326 renal carcinoma cells for subsequent analysis. We comprehensively evaluated the expression of *LHFPL2* and its relationship with clinical features, tumor prognosis, immune infiltration, and mutations. Additionally, we further assessed the correlation between *LHFPL2* and macrophage M2 polarization using single-cell data and explored its potential as a cancer therapeutic target through molecular docking. The results demonstrated that *LHFPL2* is upregulated in RCC and associated with poor survival rates. In clinical staging, the proportion of malignant and high-metastasis patients was higher in the high-*LHFPL2* group than in the low-*LHFPL2* group. Furthermore, we found that *LHFPL2* influences RCC immune infiltration, with its expression positively correlated with various immune checkpoint and M2-related gene expressions, positively associated with M2 macrophage infiltration, and negatively correlated with activated NK cells. Moreover, *LHFPL2* showed specific expression in macrophages, with the high-expression subgroup exhibiting higher M2 polarization, hypoxia, immune evasion, and angiogenesis scores, promoting tumor progression. Finally, we predicted several potential drugs targeting *LHFPL2*, such as conivaptan and nilotinib. Our analysis elaborately delineates the immune characteristics of *LHFPL2* in the tumor microenvironment and its positive correlation with macrophage M2 polarization, providing new insights into tumor immunotherapy. We also propose potential FDA-approved drugs targeting this gene, which should be tested for their binding effects with *LHFPL2* in future studies.

## 1. Introduction

Tumors are currently one of the major causes of death, and the tumor microenvironment (TME) plays a crucial role in the process of tumor development. Tumor-associated macrophages (TAMs), as one of the most abundant immune cells in the TME, often exhibit immunosuppressive tumor-promoting phenotypes when genetic alterations occur in tumors, thereby promoting tumor progression, metastasis, and the development of drug resistance [1]. The proportion of TAMs varies in different malignant tumors, with some showing lower infiltration rates, such as glioblastoma, which is defined as a “cold” tumor due to its low immune cell content [2]. Conversely, some tumors exhibit higher macrophage infiltration. Multiple studies indicated that macrophages are the most common immune cells in renal cell carcinoma [3,4,5,6]. Consequently, targeting TAMs has become a strategy in cancer therapy.

Currently, there are many approaches to tumor therapy focusing on TAMs, mainly divided into two categories: inhibiting pro-tumor TAMs, including inhibiting TAM recruitment and depleting TAMs, and activating anti-tumor TAMs, reprogramming pro-tumor macrophages into anti-tumor macrophages [7]. For example, blocking CSF1/CSF1R signaling or increasing Tregs activity in the TME directly depletes TAMs. However, the depletion of macrophages is disadvantageous for maintaining tissue homeostasis, making this treatment less effective. Similarly, drugs that block the CCL2/CCR2 pathway to prevent macrophage recruitment to tumors have not shown clinical efficacy similar to preclinical models in clinical trials [8]. The drawback of depleting TAMs is the loss of their potential immunostimulatory effects as primary phagocytic cells and specialized antigen-presenting cells within tumors. Therefore, reprogramming TAMs into an anti-tumor phenotype may be a more effective immunotherapy strategy [9]. Currently, whether polarizing TAMs from their original phenotype to an anti-tumor phenotype or targeting specific TAM functions such as phagocytosis and immune suppression, although there has been some progress in preclinical and clinical trials, there are limitations that need to be addressed [1]. New targets for TAM activation need to be explored to find new treatment opportunities.

TAMs mainly exhibit the M2-like phenotype, playing a role in promoting tumor development. This study aims to identify new M2-related targets and systematically explore the role of these genes in tumors by integrating clinical data, sample information, and tumor-related gene sets. Potential M2 polarization-related genes were screened using the differential gene expression analysis of sequencing data, among which *LHFPL2* was identified. This gene encodes a transmembrane protein and belongs to the lipoma HMGIC fusion partner (LHFP) gene family along with LHFP and *LHFPL*1, 3, 4, and 5 [10]. Since its discovery, only a few studies have confirmed that members of this family may be related to human hearing disorders [11,12] and renal developmental abnormalities [13], with limited research available. Therefore, we further analyzed its expression and prognosis in tumors, evaluated its potential as a tumor prognostic factor, and assessed its relationship with M2 macrophage polarization, investigating the mechanism by which *LHFPL2* affects tumor progression, thus providing new insights into tumor treatment directions.

## 2. Results

### 2.1. LHFPL2 Upregulation and Clinical Correlation Analysis in KIRC

**Figure 1:**
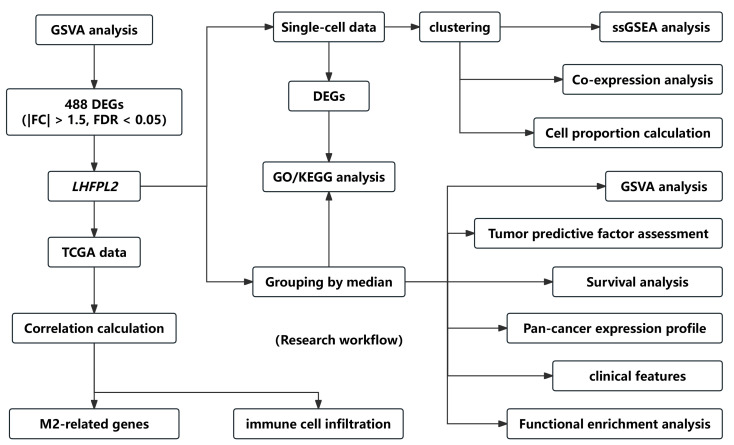
Flow chart of the research. The workflow diagram illustrates the specific process of screening the LHFPL2 gene and the subsequent research conducted on this gene.

**Figure 2:**
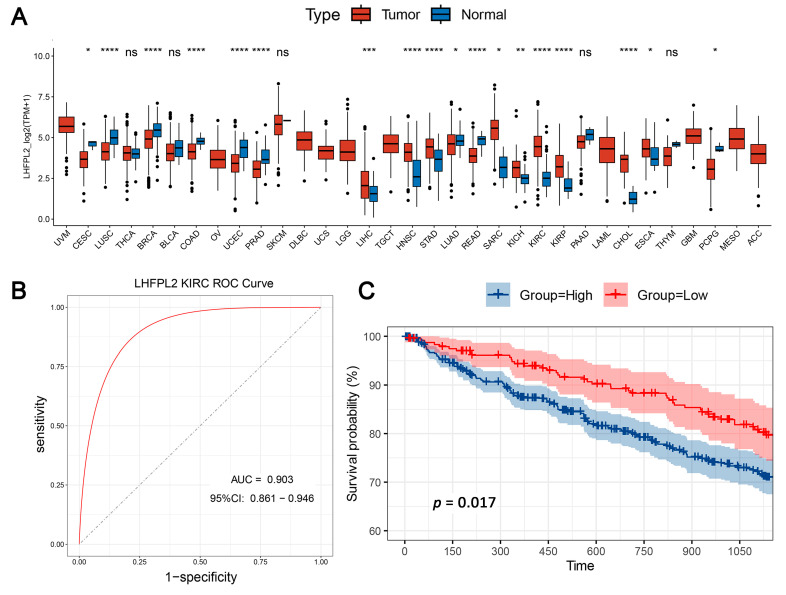

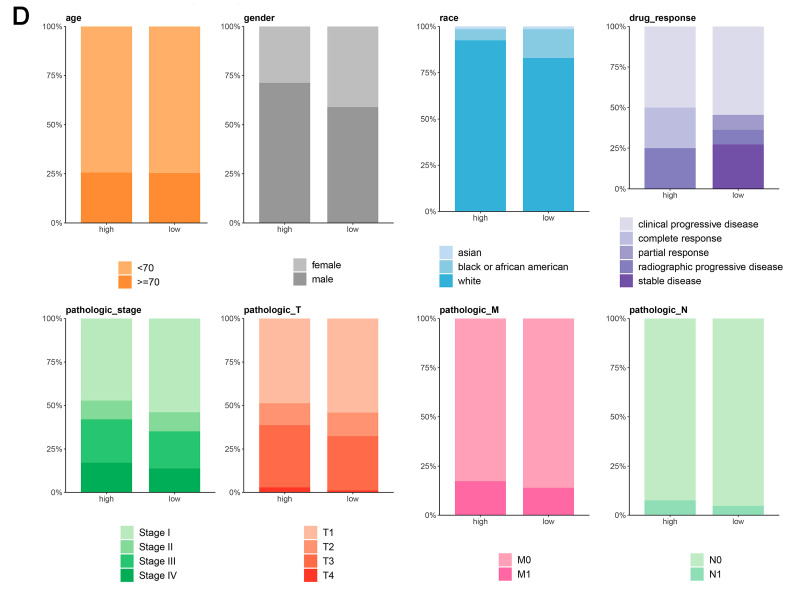
Comprehensive analysis of *LHFPL2* in cancer: expression, predictive capability, survival, and clinical correlations. (**A**) Sequencing data from tumor and normal tissues of patients with 33 types of cancer in the TCGA were used to analyze the expression profile of *LHFPL2* in human tumors and normal tissues. Box plots displayed the transcript levels of *LHFPL2* across various cancers, and the significance differences were calculated using the Wilcoxon Test. * *p* < 0.05, ** *p* < 0.01, *** *p* <0.001, **** *p* < 0.0001, ns, not significant. (**B**) The AUC value represents the predictive capability of *LHFPL2* for KIRC. The larger the AUC value, the stronger the predictive ability for the tumor. The red curve in the figure represents the ROC curve. The black dashed line indicates the performance of a random guess classifier. If the model's ROC curve is above this line, it indicates that the model has better discriminative ability than random guessing. (**C**) Survival curves were plotted with OS as the ordinate to demonstrate the impact of *LHFPL2* expression on the survival rate of KIRC patients. The log-rank test was used to assess the differences between the two groups. *p* < 0.05 indicates a significant difference. (**D**) Clinical correlations associated with *LHFPL2* expression levels in KIRC. The vertical axis of the bar chart represents the proportion of patients, while the horizontal axis represents the high and low groups of *LHFPL2*.

The overall workflow of this study is illustrated in Figure 1. The proportion of macrophages varies across different malignant tumors. Given the heightened enrichment of macrophages in liver [3,14], renal [3,4,6], and gastric cancers [15], we designated the Gene Set Variation Analysis (GSVA) score for the macrophage M2 polarization-related gene set as the M2 score. We stratified cancer samples into high and low groups based on the median M2 score, then observed the expression levels of various M2-related genes in each group, identifying differential genes and singling out *LHFPL2* as a target gene (Supplementary Figure S1). The analysis of sequencing data and clinical information from The Cancer Genome Atlas (TCGA) revealed significant upregulation of *LHFPL2* transcription levels in certain cancers compared to normal samples, particularly evident in kidney renal clear cell carcinoma (KIRC) (Figure 2A). Receiver Operating Characteristic (ROC) curves were employed to evaluate the potential as a tumor-predictive factor of LHFPL2. The Area Under the Curve (AUC) values for *LHFPL2* upregulation across five tumor types were calculated, and the AUC values are consistently higher than a random value (AUC > 0.5). Notably, the highest AUC value for *LHFPL2* was observed in KIRC, reaching 0.903 (Figure 2B and Figure S2). Furthermore, Kaplan–Meier analysis illustrated a significant association between elevated LHFPL2 expression and overall survival (OS) in KIRC, where patients with high LHFPL2 expression exhibited significantly poorer prognosis (*p* = 0.017, Figure 2C), contrasting with the absence of similar correlations in the other four cancer types (Supplementary Figure S3). Therefore, our emphasis will be on investigating the role of the LHFPL2 gene in KIRC.

The pathological stage of a tumor is a crucial indicator for determining treatment plans, predicting prognosis, and conducting clinical trials for cancer patients. By analyzing the proportion of different tumor stages within the high and low *LHFPL2* expression groups, we explored the relationship between gene expression and various aspects of tumors, such as size, lymph node involvement, and metastasis, to assess whether *LHFPL2* influences tumor progression. The results indicate that the high *LHFPL2* expression group exhibited a higher proportion of advanced-stage tumors (stage III and IV), T4 tumors, M1 tumors, and N1 tumors, while the low *LHFPL2* expression group had a higher proportion of patients with stable disease (Figure 2D). This suggests a potential association between high *LHFPL2* expression and tumor progression and aggressiveness, while low expression may correlate with a more stable tumor status. Clinical characteristics of KIRC patients are detailed in Supplementary Table S1.

### 2.2. LHFPL2 Is Involved in Multiple Tumor-Related Pathways and Immune Infiltration in KIRC

To investigate how *LHFPL2* affects tumor progression, the fifty hallmark gene sets from the MiSgDB database were utilized to calculate GSVA scores on each sample from five cancers. The results demonstrate that the majority of pathways in the hallmark gene sets exhibit higher GSVA scores in the high-*LHFPL2* expression group and lower scores in the low-*LHFPL2* expression group (Supplementary Figure S4A). Moreover, most of these scores show a significant positive correlation with *LHFPL2* expression levels (Supplementary Figure S4B). These pathways predominantly include cancer-related processes such as angiogenesis, hypoxia, and epithelial–mesenchymal transition. The upregulation of these scores in the high *LHFPL2* expression group suggests that under conditions of high-*LHFPL2* expression, numerous pro-carcinogenic pathways are altered, potentially implicating *LHFPL2* in tumor proliferation and immune response processes.

**Figure 3:**
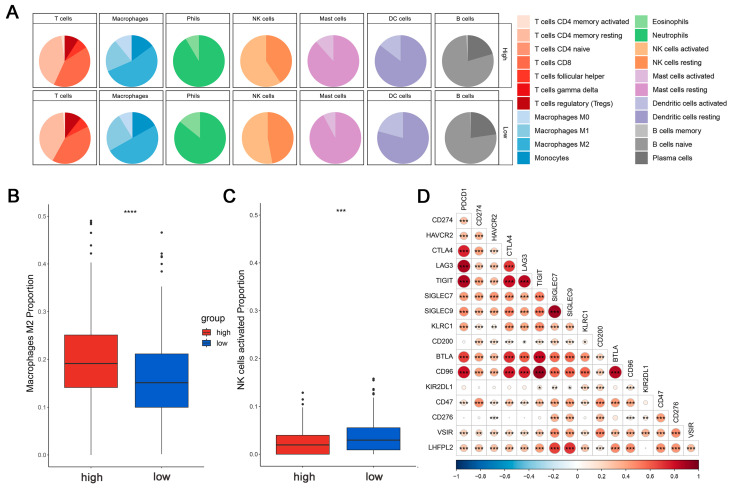

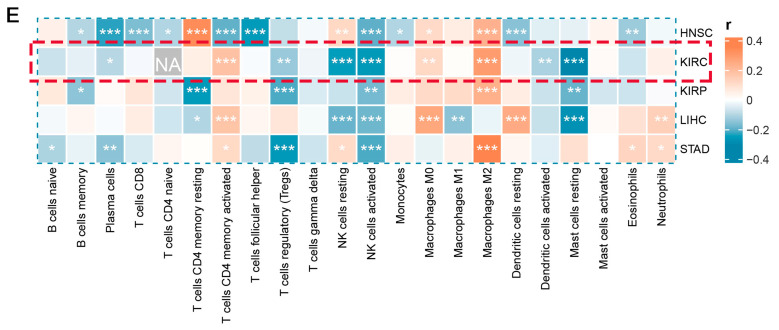
Immune infiltration profiles of *LHFPL2*. (**A**) Abundance differences of six major subtypes of immune cells between high and low expression groups of *LHFPL2* in KIRC. The area of the sectors represents the proportion of immune cell infiltration. (**B**,**C**) Variations in the proportion of M2 macrophages (**B**) and activated NK cells (**C**) between high and low expression groups of *LHFPL2* in KIRC. *** *p* <0.001, **** *p* < 0.0001. (**D**) Correlation between *LHFPL2* expression and the expression levels of various immune checkpoint markers in KIRC. The size and color intensity of the circles represent the correlation coefficients. Larger circle areas and deeper colors indicate stronger correlations between two genes. Positive correlations are shown in red, while negative correlations are shown in blue. * *p* < 0.05, ** *p* < 0.01, *** *p* < 0.001. (**E**) Heatmap illustrating the correlation between *LHFPL2* expression levels and the proportions of 22 types of immune cells in KIRC. The depth of color represents the magnitude of the correlation coefficient, with positive correlation indicated by orange and negative correlation indicated by light blue. The significance differences were calculated using the Wilcoxon Test. * *p* < 0.05, ** *p* < 0.01, *** *p* <0.001; no asterisk indicates no significant correlation. NA represents missing data, and the red dashed box highlights the renal cell carcinoma we are studying.

This is crucial for developing new cancer immunotherapy strategies to understand immune cell infiltration. The proportions of 22 immune cell types were calculated to assess the immune characteristics of the *LHFPL2* gene in the tumor microenvironment. We analyzed the proportions of each immune cell type in the high and low *LHFPL2* expression groups. Results revealed that in KIRC, the LHFPL2 high-expression group has a higher proportion of M2 macrophages (Z = 5.714, *p* = 2 × 10^−9^) and a lower proportion of activated NK cells (Z = −3.603, *p* = 0.00012) compared to the LHFPL2 low-expression group (Figure 3A–C). Correlation analysis showed a significant positive correlation between *LHFPL2* expression levels and M2 macrophage abundance in KIRC, while a significant negative correlation was observed with the abundance of activated and resting NK cells (Figure 3E). Furthermore, the correlation between *LHFPL2* and 16 immune checkpoints was examined, and the relationship between *LHFPL2* and immune responses was assessed. Statistically significant correlations were found between *LHFPL2* and other immune checkpoints, except for KIR2DL1 (Figure 3D). The correlation coefficients ranged from 0.15 to 0.71, with the highest correlation observed with SIGLEC7/9. These receptors are highly expressed in innate immune cells, particularly macrophages, facilitating immune suppression in the tumor microenvironment.

### 2.3. The Expression Level of LHFPL2 Is Positively Correlated with the M2 Macrophages in KIRC

**Figure 4:**
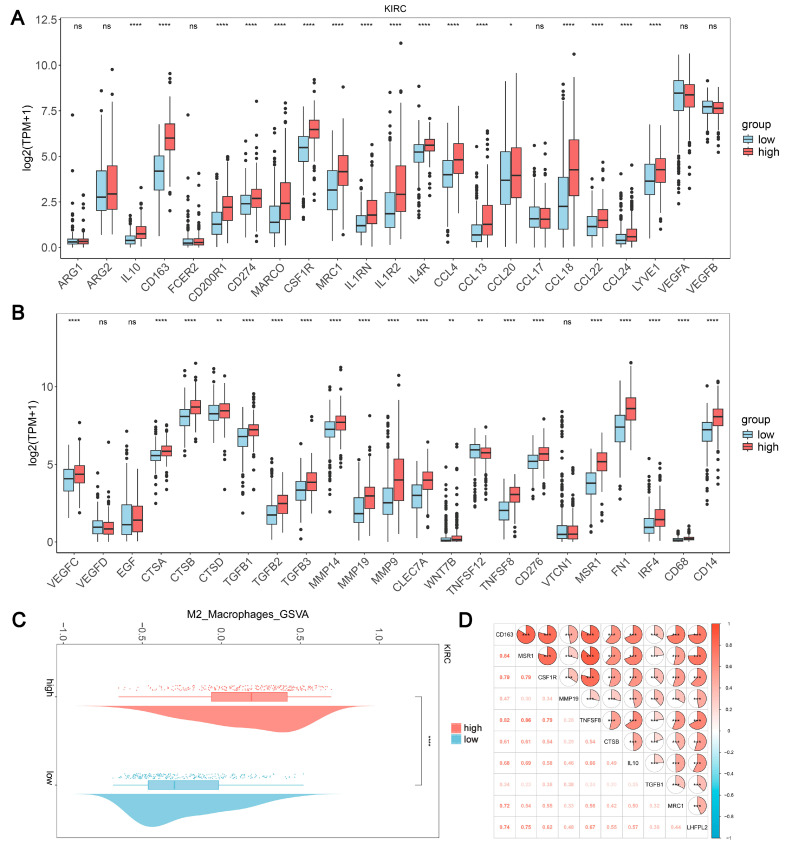
Association of *LHFPL2* expression levels with M2 macrophage. (**A**,**B**) Expression levels of M2-associated genes in *LHFPL2* high/low-expression groups. The significance differences were calculated using the Wilcoxon Test. * *p* < 0.05, ** *p* < 0.01, **** *p* < 0.0001, ns, not significant. (**C**) Differences in M2 scores between *LHFPL2* high/low-expression groups. **** *p* < 0.0001. (**D**) The correlation coefficient between the expression levels of *LHFPL2* and M2-related genes. *** *p* < 0.001. The numbers in the figure represent correlation coefficients. The size of the sectors indicates the magnitude of the correlation coefficients. Positive correlations are shown with red shading, while negative correlations are shown with blue shading. The darker the color, the stronger the correlation.

Based on the results of the immune infiltration analysis, we further explored the relationship between the expression levels of *LHFPL2* and the infiltration ratio of M2 macrophages. In KIRC, we observed significant differences in the expression levels of 37 genes between the high and low expression groups of *LHFPL2*, with 36 genes showing higher expression in the high group compared to the low group (Figure 4A,B). Additionally, the M2 scores were significantly higher in the *LHFPL2* high-expression group (Figure 4C). Correlation analysis revealed a positive correlation between *LHFPL2* and M2 genes, with correlation strengths ranging from weak to strong (Figure 4D). These results collectively indicate an association between *LHFPL2* and characteristic genes of M2 macrophages, suggesting that *LHFPL2* may influence tumor progression by affecting the expression of M2 genes. This underscores the potential of *LHFPL2* as a marker for M2 macrophage-related genes.

**Figure 5:**
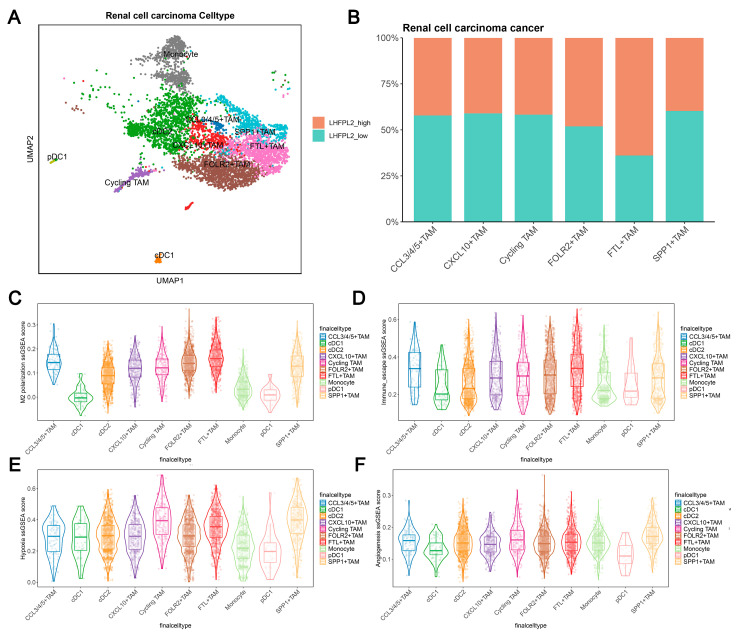
Single-cell data analysis reveals the association between *LHFPL2* gene and M2 polarization in renal clear cell carcinoma. (**A**) UMAP visualization of myeloid cells in renal cell carcinoma patients. Myeloid cells are divided into 10 clusters, including various dendritic cells (DCs), tumor-associated macrophages (TAMs), and monocytes, which are distinguished by different colors. (**B**) Proportions of high- and low-*LHFPL2*-expressing cells in six TAM subclusters. (**C**–**F**) ssGSEA scores of M2 polarization (**C**), immune escape (**D**), hypoxia (**E**), and angiogenesis (**F**) in TAM subclusters.

Cell clustering analysis was performed to examine the expression of *LHFPL2* in each cell cluster by using single-cell transcriptome sequencing data and integrating cell subtype gene sets. Additionally, single-sample gene set enrichment analysis (ssGSEA) scores for macrophage M2 polarization, hypoxia, and immune escape were utilized to assess the relationship between *LHFPL2* and macrophage M2 polarization. The results demonstrate that renal cell carcinoma can be divided into six cell types, with LHFPL2 exhibiting specific expression in myeloid lineage cells and minimal expression in other cell types (Figure 5A and Figure S5A). The myeloid cells were isolated separately and subdivided into 10 subclusters based on different marker genes. Compared to monocytes and dendritic cells, LHFPL2 showed specific expression in TAMs (Supplementary Figure S5B). Notably, the FTL+ TAM subcluster exhibited the highest expression levels of *LHFPL2*, with a higher proportion of *LHFPL2* high-expressing cells. Additionally, this subcluster showed the highest scores for M2 polarization and immune evasion while also displaying relatively high scores for hypoxia and angiogenesis (Figure 5B–F).

The TCGA and single-cell data were divided into two groups based on the expression levels of *LHFPL2*, followed by differential gene expression analysis. The upregulated and downregulated genes were then subjected to GO and KEGG analyses separately. The results revealed the enrichment of immune-related pathways among the upregulated and downregulated genes in both datasets, elucidating the association between *LHFPL2* and tumor immunity (Supplementary Figures S5C–F and S6).

### 2.4. LHFPL2 Is Related to Novel Genomic Alterations in KIRC

**Figure 6:**
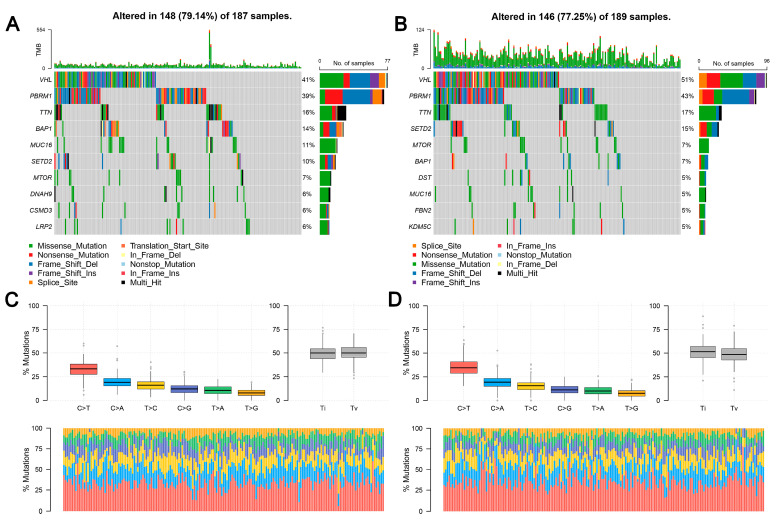

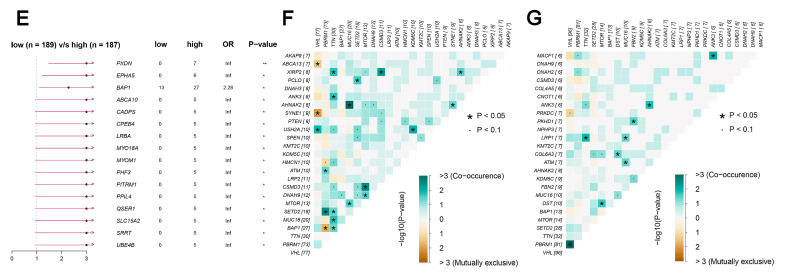
Genomic alterations of different *LHFPL2* expression groups. (**A**,**B**) The waterfall plot illustrates the top 10 frequently mutated genes in high- (**A**) and low-*LHFPL2* (**B**) expression groups in KIRC. Genes are arranged in descending order based on mutation frequency, and mutations are categorized and labeled with different colors. (**C**,**D**) The box plot illustrates the overall distribution of six different transformations in high- (**C**) and low-*LHFPL2* (**D**) expression groups, presented as stacked bar charts to display the conversion ratios in each sample. (**E**) The forest plot displays the top 16 significantly mutated genes in the high-*LHFPL2* group relative to the low-*LHFPL2* group. OR represents the ratio of mutation frequencies between the high- and low-LHFPL2 expression groups. “Inf” indicates infinity. * *p* < 0.05, ** *p* < 0.01. (**F**,**G**) The heatmap displays the co-occurrence or mutual exclusivity of 25 mutated genes between the high- (**F**) and low (**G**)-*LHFPL2* groups. Green and brown represent the probabilities of two genes undergoing either co-occurrent mutations or mutually exclusive mutations, with darker colors indicating higher probabilities.

TCGA data reveal that the frequency of *LHFPL2* SNV is close to zero in the KIRC. Therefore, we focused on *LHFPL2*-related changes in high- and low-*LHFPL2* groups instead of exploring *LHFPL2*’s self-genomic alterations. A comparison of SNV profiles between the high- and low-*LHFPL2*-expression groups is depicted in waterfall plots (Figure 6A,B). Missense mutations were found to be the predominant mutation type in both high- and low-*LHFPL2* expression groups (Supplementary Figures S7 and S8). Additionally, the mutation status of genes in both groups was analyzed. It was observed that there were significant genomic alterations in both groups, with genes like *VHL*, *PBRM1*, and *TTN* exhibiting higher mutation rates (Figure 6A–D). However, genes like *PXDN*, *EPHA5*, *BAP1*, *ABCA10*, and *CADPS* showed relatively higher mutation frequencies in the high-LHFPL2-expression group (Figure 6E). Heatmap results also indicated that the strongest co-occurring gene mutation pair in the high-*LHFPL2* expression group was *AHNAK2*-*MUC16*, while the strongest mutually exclusive gene mutation pair was *SYNE1*-*VHL* (Figure 6F). Conversely, the strongest co-occurring gene mutation pair was *PBRM1*-*VHL* in the low *LHFPL2* expression group (Figure 6G).

### 2.5. Molecular Docking Landscape against LHFPL2 and Prediction of Potential Drugs

**Figure 7:**
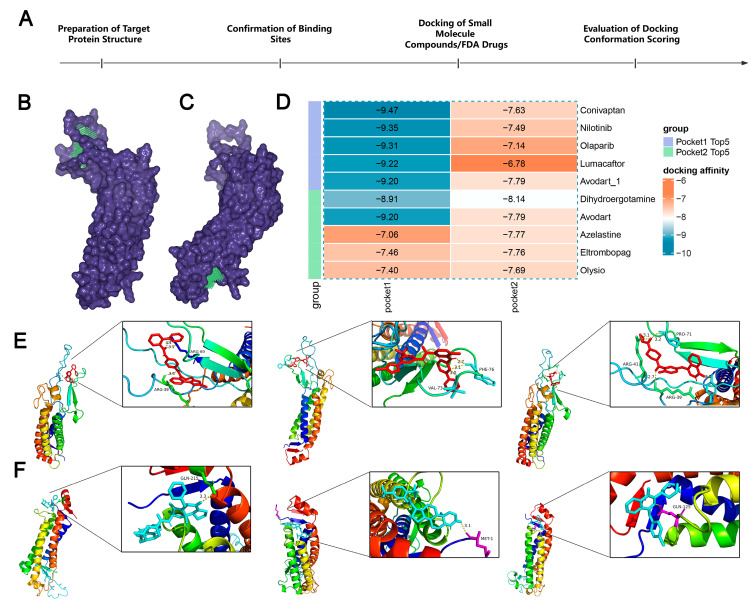
The screening of FDA-approved drugs. (**A**) Flowchart of drug screening. (**B**,**C**) *LHFPL2* protein-binding pocket 1 (**B**) and pocket 2 (**C**) predicted by DS. The blue-purple section represents the predicted protein structure of *LHFPL2*, while the green section represents the predicted binding site. (**D**) Heatmap illustrating the docking affinity scores between the *LHFPL2* protein and FDA-approved drugs. The lower the affinity score, the stronger the affinity. (**E**) The molecular docking results of the top three FDA-approved drugs with the highest affinity scores for docking with pocket 1, labeled from left to right as Conivaptan, Nilotinib, and Olaparib. (**F**) The molecular docking results of the top three FDA-approved drugs with the highest affinity scores for docking with pocket 2, labeled from left to right as Dihydroergotamine, Avodart, and Azelastine. The dashed lines represent hydrogen bonds, the numbers indicate the hydrogen bond lengths, and the hydrogen bonds connect the drug to the amino acid residues on the protein.

To further understand the potential of LHFPL2 as a drug target, we docked the protein structure with over 800,000 compounds and 1614 FDA-approved drugs to screen for compounds with inhibitory potential against LHFPL2 (Figure 7A). Based on the predicted structure of LHFPL2 protein, six molecular docking pockets were identified using Discovery Studio software. The two largest pockets were selected for subsequent docking operations, named Pocket 1 and Pocket 2 (Figure 7B,C). Subsequently, UniDock was used to dock LHFPL2 with a dataset of one million compounds from the zinc compound library and 3000 drugs from the FDA database, calculating the affinity scores between the protein and different ligands. Lower docking scores indicate stronger binding between compounds and the protein. Results revealed that, for Pocket 1, FDA drugs Conivaptan (vasopressin receptor antagonist), Nilotinib (Bcr-Abl tyrosine kinase inhibitor), and Olaparib (PARP inhibitor) had the highest affinity scores, with values of −9.47, −9.35, and −9.31, respectively (Figure 7D). Among compounds, subTS_HIT102125848, subTS_HIT104720933, and subTS_HIT101532189 exhibited the highest affinity scores, with values of −12.48, −11.69, and −11.55, respectively (Supplementary Figure S9A). For Pocket 2, FDA drugs Dihydroergotamine, Avodart, and Azelastine had the highest affinity scores, with values of −8.14, −7.79, and −7.77, respectively (Figure 7D), while compounds subTS_HIT213615638, subTS_HIT213008249, and subTS_HIT213824420 had the highest affinity scores, with values of −9.65, −9.48, and −9.44, respectively (Supplementary Figure S9A). The binding poses and sites are shown in Figure 7E,F and Supplementary Figure S9B,C. The interaction between the *LHFPL2* protein and compounds or FDA-approved drugs was also depicted with a 2D diagram (Supplementary Figure S10).

## 3. Materials and Methods

### 3.1. The Collection and Processing of Sequencing and Clinical Data

The transcriptome data utilized in this study were obtained from The Cancer Genome Atlas (TCGA) database (https://www.cancer.gov/ccg/research/genome-sequencing/tcga, accessed on 1 April 2024), comprising TPM and counts data, tumor staging, as well as patient demographics including age, gender, and overall survival time across 33 cancer types. Single-cell data for renal cell carcinoma were acquired from the SingleCellPortal platform (https://singlecell.broadinstitute.org/, accessed on 1 April 2024), originating from studies by Wu SZ [16] and Bi K [17], among others. The mutation data used for SNP analysis also originate from the TCGA database.

### 3.2. Differential Gene Expression in Cancer and Clinical Feature Analysis

Gene expression in tumor and normal tissues from TCGA were analyzed using the “ggplot” (version 3.3.5) and “ggpubr” (version 0.4.0) packages of R (version 4.3.1). A comparison was conducted between the two groups. Pathological staging refers to the severity assessment of tumors based on tissue samples obtained from surgery or biopsy, categorized into stages I through IV. The American Joint Committee on Cancer (AJCC) classifies cancer based on the size and extent of the primary tumor (T0, T1, T2, T3, T4), regional lymph node involvement (N0, N1, N2, N3), and the presence of distant metastasis (M0, M1). Tumors are classified based on their response to drugs into clinical progressive disease, complete response, partial response, radiographic progressive disease, and stable disease. Analyzing the proportion of different statuses in the *LHFPL2*-high and -low groups according to these classification criteria reveals the relationship between *LHFPL2* and the clinical characteristics of tumors. Specific classification information is sourced from https://link.springer.com/chapter/10.1007/978-1-4614-2080-4_1 (accessed on 1 April 2024).

### 3.3. Gene Set Variation Analysis (GSVA) and Single-Sample Gene Set Enrichment Analysis (ssGSEA)

The calculation of GSVA and ssGSEA was performed using packages such as “GSVA” (version 1.40.1), “GSEABase” (version 1.55.1), “msigdbr” (version 7.4.1), “limma” (version 3.36.5), “Seurat” (version 5.1.0), and “readr” (version 2.1.5) in the R software (version 4.3.1). The gene sets used in this study are detailed in Supplementary Table S2. The specific calculation formula for GSVA scores is as follows, where *X*_ik_ represents the expression level of gene “*k*” in sample “*i*”. Mean(*S*_.j_) denotes the mean expression level of gene set “*j*” across all samples, and *sd*(*S*_.j_) indicates the standard deviation of gene set “*j*” across all samples.

$${S^{\prime }}_{\mathrm{ij}}=\frac{\sum_{k\in geneset_{j}}X_{ik}-mean(S_{.j})}{sd(S_{.j})}$$

The ssGSEA score is calculated using the following formula, where *ES*(*g*,*S*) is the enrichment score of gene set “*S*” on gene “*g*”, *Rank*(*g*,*i*) is the rank of gene g in sample “*i*”, “*w*_i_” is the weight of sample “*i*”, and “*n*” is the number of samples:

$$ES(g,S)=\frac{\sum_{i=1}^{n}Rank(g,i)\times w_{i}}{\sum_{i=1}^{n}w_{i}}$$

### 3.4. Assessment of Tumor Predictive Factors and Survival Analysis

Using the “pROC” (version 1.18.5) package in R software (version 4.3.1), the expression level of *LHFPL2* and sample tissue type (tumor, normal) were taken as input values. With a confidence interval of 95%, the direction parameter in the roc() function was set to determine the direction of the ROC curve. The ROC curve was then plotted, and the AUC value was calculated. The formula for calculating the AUC is as follows: where “x_i_” and “y_i_” are the coordinates of the i-th point on the ROC curve, and “n” is the total number of points on the ROC curve.

$$AUC=\sum_{i=1}^{n-1}\frac{{(x}_{i+1}{-x}_{i}{)(y}_{i}+y_{i+1})}{2}$$

Based on evaluation criteria, the predictive value of the model was assessed. TCGA data on *LHFPL2* expression levels and clinical information were processed. Using the “survival” (version 3.6.4) and “survminer” (version 0.4.9) packages on Hiplot website (https://hiplot.cn/, accessed on 1 April 2024), the Kaplan–Meier method was applied to estimate patient survival rates and plot survival curves. The specific formula for the survival rate in the survival curve is as follows: “*t_i_*” is the *i*-th event time point, “*d_i_*” is the number of events occurring at time “*t_i_*”, and “*n_i_*” is the number of individuals still under study at time “*t_i_*”.

$$S(t_{i})=S(t_{i}-1)(1-\frac{d_{i}}{n_{i}})$$

The log-rank test was utilized to compare the survival differences between the high- and low-*LHFPL2* expression groups. The calculation formula is as follows: where “*O*” is the sum of observed event counts (e.g., death events), and “*E*” is the sum of expected event counts, calculated based on the overall risk of the two or more groups of survival data. A *p*-value less than 0.05 indicates statistical significance in the difference [18].

$$\chi^{2}=\frac{{\left[O-E\right]}^{2}}{E}$$

### 3.5. Immune Infiltration Analysis

The details of the immune checkpoint molecules used in this study are provided in Table 1, including the names of the factors and their specific sources. The gene feature matrix from the “Cibersort” (https://cibersortx.stanford.edu/, accessed on 7 April 2024) was utilized for the investigation of immune cell infiltration proportions, containing 547 genes capable of distinguishing 22 human cell phenotypes [19]. Based on this matrix, the infiltration proportions of 22 immune cells were calculated.

### 3.6. Correlation Analysis

Spearman’s rank correlation coefficient “r” is calculated using the following formula: where “*d*” is the difference between the ranks of corresponding variables, and “*n*” is the number of observations.

$$r=1-\frac{6\sum d^{2}}{n(n^{2}-1)}$$

The strength of correlation is classified as follows: a correlation coefficient greater than 0.7 indicates a strong correlation, between 0.5 and 0.7 indicates a moderate correlation, between 0.3 and 0.5 indicates a weak correlation, and a correlation coefficient less than 0.3 suggests no correlation. The Spearman rank correlation coefficient assessed the relationship between different datasets using the cor.test() function. The statistical significance of correlation is determined by the size of the *p*-value. The specific formula is as follows: where “r” represents the Spearman correlation coefficient and “n” denotes the sample size.

$$p=\frac{r}{\sqrt{\frac{1-r^{2}}{n-2}}}$$

### 3.7. Single-Cell Data Analysis

Seurat (version 5.1.0) is a toolbox for single-cell data analysis, used for cell clustering and differential analysis. After creating a Seurat object, low-quality cells are filtered based on the number of genes and the proportion of mitochondrial genes. Counts are then normalized, and highly variable genes between cells are selected for subsequent clustering, followed by normalization and dimensionality reduction. In this study, a manual annotation approach was employed, combining specific genes and gene sets collected from the literature for each cell cluster, annotating cell clusters based on the expression of genes in different clusters [27]. Additionally, differential expression analysis was conducted between the high- and low-expression groups of *LHFPL2* in TAMs using the FindMarkers function, with a threshold of 0.25.

### 3.8. Functional Enrichment Analysis

In this study, functional enrichment analysis was conducted by using two databases: Gene Ontology (GO) and Kyoto Encyclopedia of Genes and Genomes (KEGG). The GO enrichment pathway analysis was divided into three parts: Biological Process (BP), Cell Component (CC), and Molecular Function (MF). Differential expression genes identified in our study were analyzed for functional enrichment using the “clusterProfiler” (version 3.18.1) package in R software (version 4.2.1), and the findings were visualized using Hiplot website (https://hiplot.cn/, accessed on 1 April 2024).

### 3.9. Single-Nucleotide Variant Analysis

The single-nucleotide variant (SNV) data for KIRC in TCGA was downloaded from the GDC data portal using the R package “TCGAbiolinks” (version 2.18.0). After merging and organizing the information with the grouping of target genes, the R package “maftools” (version 2.10.1) was utilized to visualize the SNP profiles in different groups.

### 3.10. Compound and FDA-Approved Drug Analysis

The predicted structure of the LHFPL2 protein was obtained from the AlphaFold protein structure database (https://alphafold.ebi.ac.uk/, accessed on 1 April 2024). Compounds for molecular docking, as well as FDA-approved drugs, were downloaded from the Topscience database and the ZINC20 database (https://zinc.docking.org/, accessed on 1 April 2024), respectively. Binding site prediction was performed using Discovery Studio software (version 4.5). Preprocessing of protein structures and ligands was conducted using Autodock software (version 4.1) and OpenBabel software (version 2.3.1), respectively. Molecular docking was performed using UniDock software (version 1.1), with visualization of docking results carried out using PyMOL (version 2.5.7).

### 3.11. Statistical Analysis

All statistical analyses were conducted using R software (version 4.2.1). Spearman correlation coefficient was employed for correlation analysis. The Wilcoxon Test was utilized to compare the differences between any two groups of continuous variables. Kaplan–Meier survival analysis was conducted to evaluate the prognostic significance of *LHFPL2* expression. Survival curves were compared using the log-rank test to determine statistically significant differences between groups. A significance level of *p* < 0.05 was considered statistically significant and denoted by an asterisk (*). Specifically, * indicated *p* < 0.05, ** indicated *p* < 0.01, and *** indicated *p* < 0.001, representing different levels of significance.

## 4. Discussion

RCC, a malignant tumor originating from renal tubular epithelial cells [28], accounts for about 85% of all malignant kidney tumors and significantly impacts human health. Current treatments benefit few patients, underscoring the need for effective therapies [29]. TAMs in TME, mainly M2 phenotype, play a major role in tumor progression and immune evasion [30,31,32]. Their immunosuppressive and tumor-promoting effects hinder RCC therapy. Targeting TAMs to modulate their phenotype and function may lead to new therapeutic strategies. This study identified a novel M2 macrophage-related gene, *LHFPL2*, by calculating the GSVA score of M2 macrophages. We analyzed its clinical characteristics and computed its significant correlations with the abundance of various immune cells, expression levels of M2 marker genes, immune checkpoint levels, and tumor-related pathways by using gene expression matrices from 33 tumor types in the TCGA database and clinical information from RCC tumor samples. These findings were further validated using single-cell transcriptome data, thereby confirming the potential of this gene as a therapeutic target for treating RCC associated with M2 macrophages.

The upregulation of LHFPL2 is associated with clinical characteristics and tumor progression. We observed that *LHFPL2* expression is significantly upregulated in some tumor tissues such as renal cancer, liver cancer, gastric cancer, and head and neck squamous cell carcinoma, demonstrating good predictive ability in these cancers, though it cannot yet serve as a prognostic factor for cancers other than RCC. The specificity of *LHFPL2* in RCC may be related to its function. For instance, *LHFPL5*, another protein in the LHFP gene family and the most studied one has been shown to be associated with kidney dysplasia in boys when disrupted [13]. This suggests that the *LHFP* family might have a special influence on kidney development and changes. This increase is associated with poor OS in RCC patients, and most tumor samples with high-*LHFPL2* expression are at high-risk pathological stages with high metastasis rates compared to the low-*LHFPL2* expression group. We hypothesize that LHFPL2 may promote the development and poor prognosis of RCC by modulating immune infiltration within the TME.

*LHFPL2* affects immune infiltration in RCC. There are also immune cells in the body besides tumor cells. The immune system can inhibit tumor growth through mechanisms such as immune surveillance, but it can also aid tumors in achieving immune evasion through specific mechanisms. Understanding immune cell infiltration is crucial for developing new cancer immunotherapy strategies. This study confirmed the significant difference in the infiltration proportions of activated NK cells and M2 macrophages between the high- and low-*LHFPL2* expression groups by analyzing the differences in infiltration proportions and associating *LHFPL2* expression levels with these proportions. Activated NK cells primarily exert tumor immune surveillance function. They infiltrate tumor regions and are positively correlated with extended patient survival [33]. NK cells can directly eliminate cancer cells by interacting with death receptors on target cells or by secreting cytokines to directly inhibit tumor proliferation or induce apoptosis [34]. M2 macrophages promote tumor progression and exert immunosuppressive functions through various pathways, mediating the secretion of factors like VEGF and CCL18 to promote tumor angiogenesis [35]. Current research indicates that TAMs and NK cells interact to limit antitumor immunity [36,37]. In our study, *LHFPL2* expression was positively correlated with M2 macrophages and negatively correlated with activated NK cells, aligning with the existing view that these cells interact to influence antitumor responses. *LHFPL2* may play a crucial role in this reciprocal regulation, mediating tumor development. We hypothesize that *LHFPL2* might participate in tumor progression in various TME contexts by regulating different immune cells. In renal cancer, it likely influences tumor progression primarily by modulating the quantity and status of macrophages and NK cells and their interactions, thereby affecting immune suppression and promoting tumor development.

*LHFPL2*, as a gene associated with M2 polarization of macrophages, influences the progression of RCC. In this study, the analysis of immune infiltration results revealed that LHFPL2 expression is positively correlated with the infiltration proportion of M2 macrophages. To validate the relationship between *LHFPL2-* and M2-associated genes, we compared the differences in M2 scores between high- and low-*LHFPL2* expression groups, further confirming the connection between *LHFPL2* and M2 macrophage polarization. Compared to the low-*LHFPL2* expression group, the high-*LHFPL2* expression group had higher M2 scores and higher expression of M2-associated genes, with *LHFPL2* significantly positively correlated with M2-associated genes. Additionally, among the 37 genes with significantly different expression levels between the high and low groups, 36 genes were expressed higher in the high group. Moreover, *LHFPL2* was positively correlated with M2 marker genes, with correlation strengths ranging from weak to strong. This indicates that *LHFPL2* is associated with M2 macrophage genes and may influence tumor progression by affecting the expression of M2 genes. These findings were also confirmed in single-cell data analysis. The FTL+ TAM subpopulation with the highest M2 score also exhibits the highest expression levels of *LHFPL2*, with a higher proportion of cells showing high expression of *LHFPL2*. The *FTL* gene encodes a subunit of ferritin, and studies have shown its overexpression in malignant tumors such as hepatocellular carcinoma, malignant mesothelioma, and glioblastoma, which may lead to poor prognosis in patients [38,39,40]. Additionally, it is involved in the regulation of iron homeostasis in the body, responsible for iron storage and release. Single-cell data from The Human Protein Atlas database also reveals that among the top 15 genes significantly positively correlated with *LHFPL2*, besides M2 marker genes like *MS4A4A*, *CTSB*, *CD163*, and *C1QB*, it includes *HMOX1*, a protein-encoding factor that promotes both the M2 polarization of macrophages and ferroptosis [41,42]. Some studies have found that in the tumor microenvironment, ferroptosis promotes M2 polarization of macrophages by releasing iron and ROS, thereby supporting tumor growth and immune evasion [43]. Therefore, it is possible that *LHFPL2* regulates M2 polarization of macrophages by participating in the regulation of iron metabolism, which may influence factors involved in iron metabolism, such as *HMOX1* and *FTL*.

*LHFPL2* holds significant potential as a therapeutic drug target. Pharmacological inhibition of specific targets is considered a potential effective treatment strategy to prevent tumor metastasis. In this study, we screened several small-molecule drugs that may inhibit *LHFPL2*, including Nilotinib, Olaparib, and Azelastine. These drugs have been proven effective in treating various cancers [44,45,46], including renal cancer, and are used in clinical settings. Additionally, some of these drugs can modulate macrophage functions. For instance, Olaparib can reprogram TAMs, enhancing their cytotoxicity and phagocytic activity [47]. Avodart treatment can inhibit the increase in pro-inflammatory macrophage density [48]. Azelastine can inhibit macrophage chemotaxis and phagocytic activity in vitro [49]. These findings support the potential of *LHFPL2* as a viable drug target. However, the docking simulations were conducted under idealized conditions, which do not fully reflect the complexity of the intracellular environment. In real cells, macromolecular crowding and confined spaces (such as pores) significantly affect molecular interactions [50]. In the crowded environment of the cytoplasm, the presence of high concentrations of macromolecules like proteins and nucleic acids can influence the binding affinity and dynamics of drug molecules [51]. Additionally, many cellular reactions occur in confined spaces where spatial constraints can alter the conformational flexibility and accessibility of both the drug and the target protein [52,53]. Compared to bulk solutions, these restricted environments can lead to changes in binding affinities and kinetic properties. Further experimental validation is required to determine whether the protein encoded by this gene can indeed bind to the drugs we have screened, and this will be a primary focus of our future work.

As a transmembrane protein, *LHFPL2* belongs to the *LHFP* gene family [54]. It has been relatively understudied, with only a few studies highlighting its importance in reproductive tract development and infertility research [10]. Our study is the first to link *LHFPL2* with tumors and the immune microenvironment, systematically revealing the expression of *LHFPL2* in renal cell carcinoma and its significant correlation with tumor immune infiltration, especially M2 macrophage infiltration. By utilizing omics data, we validated its specific high expression in TAMs and its association with macrophage M2 polarization, demonstrating its potential as a therapeutic target for renal cell carcinoma. However, there are still some limitations in this study. Firstly, our research relies mainly on bioinformatics analysis and publicly available databases, which cover a limited range of databases and cancer types and do not involve protein or metabolic-level results. Secondly, our study is limited to renal cell carcinoma, and the role of *LHFPL2* in other types of tumors remains unclear. Lastly, the identified genes were only studied at the bioinformatics level, without corresponding wet lab experiments or in vivo validations. In the future, we aim to further evaluate the potential of *LHFPL2* as a therapeutic target for RCC related to M2 macrophages at the experimental level and develop new drugs.

## Figures and Tables

**Table 1:** The sources of immune checkpoint molecules.

Checkpoint	Sample Count	Source
PD1/PDCD1	Programmed Cell Death Protein 1	Morad et al. [20]
PDL1/CD274	Programmed Death-Ligand 1	
TIM3/HAVCR2	Hepatitis A virus Cellular Receptor 2	
CTLA4	Cytotoxic T-Lymphocyte-Associated Protein 4	
LAG3	Lymphocyte-Activation Gene 3	
VISTA/VSIR	V-domain Ig Suppressor of T Cell Activation	
TIGIT	T Cell Immunoreceptor with Ig and ITIM Domains	Rotte et al. [21]
siglecs-7/9	Sialic Acid-Binding Ig-Like Lectin 7/9	Stanczak et al. [22]
NKG2A/KLRC1	Natural Killer Group 2A	Archilla-Ortega et al. [23]
CD200	Cluster of Differentiation 200	
BTLA	B and T Lymphocyte Attenuator	
CD47	Cluster of Differentiation 47	
CD96	Cluster of Differentiation 96	Sivori et al. [24]
KIR2DL1	Killer Cell Immunoglobulin-Like Receptor, Two Domains, Long Cytoplasmic Tail, 1	Liu et al. [25]
B7-H3/CD276	B7 Homolog 3	Getu et al. [26]

## Data Availability

Data are contained within the article.

## Supplementary Materials

The following supporting information can be downloaded at: https://www.mdpi.com/article/10.3390/ijms25126707/s1.
