# Supplementary material for: LHFPL2 Serves as a Potential Biomarker for M2 Polarization of Macrophages in Renal Cell Carcinoma
Source: Int J Mol Sci. 2024 Jun 18;25(12):6707. doi: 10.3390/ijms25126707 (PMC11204190; doi:10.3390/ijms25126707)
Supplement: Supplementary file 1 [file ijms-25-06707-s001.zip › ijms-3041492-supplementary/ijms-3041492-supplementary/Supplementary Figures Captions.pdf]

LHFPL2 Serves as a Potential Biomarker for M2 Polarization of Macrophages in Renal Cell Carcinoma

Xiaocheng Gong †, Yunfei Liu †, Qian Zhang, Keying Liang, Jinfen Wei and Hongli Du *

School of Biology and Biological Engineering, South China University of Technology, Guangzhou, China
* Correspondence: hldu@scut.edu.cn
† These authors contributed equally to this work.

Suppementary Figures Captions

Supplementary Figure S1. Identification of key genes in this study. **(A)** The TCGA samples in KIRC were divided into high and low groups by using the GSVA scores of the M2 gene set. The heatmap shows the expression levels of M2-related genes in different samples within the high and low groups. The color intensity represents the log2 (TPM+1) values, with red indicating high expression and blue indicating low expression. **(B–E)** Volcano plots showing differential gene expression between high and low M2 score groups in KIRC **(B)**, KIRP **(C)**, LIHC **(D)**, and STAD **(E)**. Red dots represent genes with |Fold Change (FC)| > 1.5 and FDR < 0.05, indicating significant differential expression. Green dots represent genes with |FC| > 1.5 and FDR > 0.05. Blue dots represent genes with |FC| < 1.5 and FDR < 0.05. Gray dots represent genes with |FC| < 1.5 and FDR > 0.05. **(F)** Bar graphs showing the number of DEGs in KIRC. **(G)** Venn diagram showing the intersection of DEGs between high and low M2 score groups across four types of cancer.

Supplementary Figure S2. The AUC value represents the predictive capability of *LHFPL2* for KIRP **(A)**, STAD **(B)**, LIHC **(C)** and HNSC **(D)**. The larger the AUC value, the stronger the predictive ability for the tumor.

Supplementary Figure S3. Survival curves were plotted with OS as the ordinate to demonstrate the impact of LHFPL2 expression on the survival rate of KIRP **(A)**, STAD **(B)**, LIHC **(C)** and HNSC **(D)** patients. The log-rank test was used to assess the differences between the two groups. $p < 0.05$ indicates a significant difference.

Supplementary Figure S4. Association of *LHFPL2* Expression Levels with tumor-associated pathways. **(A)** Based on LHFPL2 expression levels, the TCGA samples in KIRC were divided into high and low LHFPL2 expression groups. The heatmap shows the GSVA scores of tumor-related pathway gene sets in different samples between the high and low groups, with color intensity representing the GSVA score levels. **(B)** Spearman correlation coefficient analysis was used to calculate the correlation between LHFPL2 expression levels and tumor-related pathway GSVA scores. The deeper the color, the stronger the correlation, with red indicating a positive correlation and blue indicating a negative correlation. The cor.test() function was used for Spearman correlation coefficient testing, with *p<0.05, **p<0.01, and ***p<0.001.

Supplementary Figure S5. Single-cell analysis of the biological characteristics and functions of *LHFPL2*. **(A)** *LHFPL2* expression across various cell types in renal clear cell carcinoma. **(B)** *LHFPL2* expression in myeloid cell subtypes in renal clear cell carcinoma. **(C-F)** Biological processes (BP) and KEGG pathways enriched with genes showing upregulation or downregulation in the high *LHFPL2* expression group within renal clear cell carcinoma.

Supplementary Figure S6. Functional enrichment analysis of DEGs in the high and low expression groups of *LHFPL2* in TCGA samples. **(A-B)** GO-BP pathway enrichment analysis **(A)** and KEGG analysis **(B)** of up-regulated genes in the LHFPL2 high expression group compared to the LHFPL2 low expression group. **(C-D)** GO-BP pathway enrichment analysis **(C)** and KEGG analysis **(D)** of down-regulated genes in the LHFPL2 high expression group compared to the LHFPL2 low expression group.

Supplementary Figure S7. The SNP landscape of data in KIRC. **(A-C)** Statistical analysis of the probabilities of each variant classification **(A)**, variant type **(B)**, and SNV class **(C)** in KIRC data. Different divisions are distinguished by different colors, and the horizontal axis represents the statistical count of mutation numbers. **(D)** Statistical count of different mutations occurring in each sample. **(E)** Statistical count of specific variant classifications occurring in all samples. **(F)** The top 10 genes ranked by mutation probability and the statistical analysis of the specific types of mutations occurring in them.

Supplementary Figure S8. The SNP landscape of the high **(A)** and low **(B)** *LHFPL2* expression groups in KIRC data.

Supplementary Figure S9. The screening of compounds. **(A)** Heatmap illustrating the docking affinity scores between the LHFPL2 protein and small molecule compounds. The lower the affinity score, the stronger the affinity. **(B)** The molecular docking results of the top three compounds with the highest affinity scores for docking with pocket 2, they are labeled from left to right as subTS_HIT102125848, subTS_HIT104720933, and subTS_HIT101532189. **(C)** The molecular docking results of the top three compounds with the highest affinity scores for docking with pocket 2, they are labeled from left to right as subTS_HIT213615638, subTS_HIT213008249, and subTS_HIT213824420. The dashed lines represent hydrogen bonds, the numbers indicate the hydrogen bond lengths, and the hydrogen bonds connect the drug to the amino acid residues on the protein.

Supplementary Figure S10. 2D diagram depicting the interaction between the *LHFPL2* protein and compounds or FDA-approved drugs. **(A)** 2D diagram depicting the top three FDA-approved drugs with the highest affinity scores for docking with pocket 1, they are labeled as Conivaptan, Nilotinib, and Olaparib from left to right. **(B)** 2D diagram depicting the top three FDA-approved drugs with the highest affinity scores for docking with pocket 2, they are labeled as Dihydroergotamine, Avodart, and Azelastine from left to right. **(C)** 2D diagram depicting the top three compounds with the highest affinity scores for docking with pocket 1, they are labeled as subTS_HIT102125848, subTS_HIT104720933, and subTS_HIT101532189 from left to right. **(D)** 2D diagram depicting the top three compounds with the highest affinity scores for docking with pocket 1, they are labeled as subTS_HIT213615638, subTS_HIT213008249, and subTS_HIT213824420 from left to right.
