# Supplementary figures and images for: LHFPL2 Serves as a Potential Biomarker for M2 Polarization of Macrophages in Renal Cell Carcinoma
Source: Int J Mol Sci. 2024 Jun 18;25(12):6707. doi: 10.3390/ijms25126707 (PMC11204190; doi:10.3390/ijms25126707)

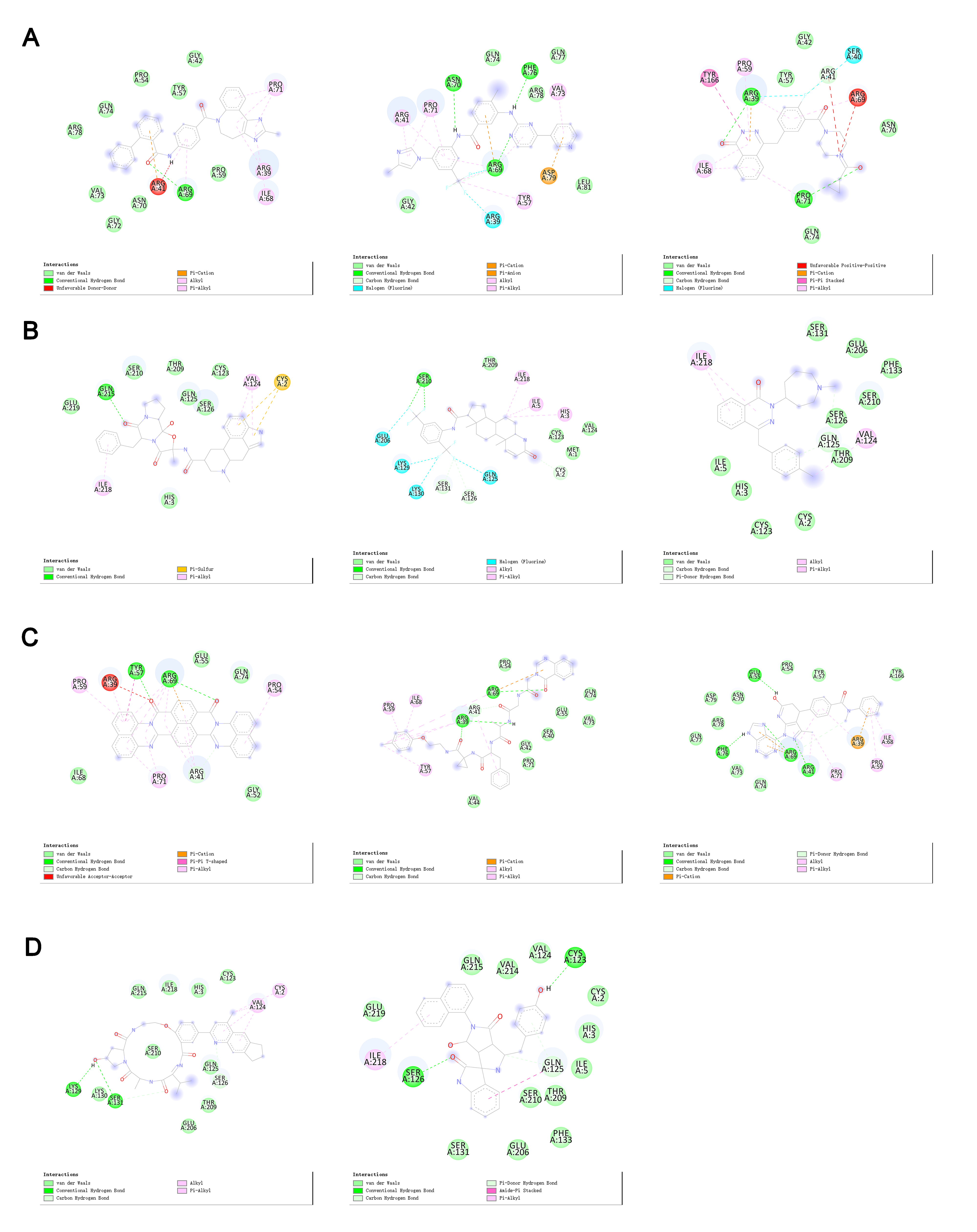

Supplement: Supplementary file 1 [file ijms-25-06707-s001.zip › ijms-3041492-supplementary/ijms-3041492-supplementary/Figure S10.tif]

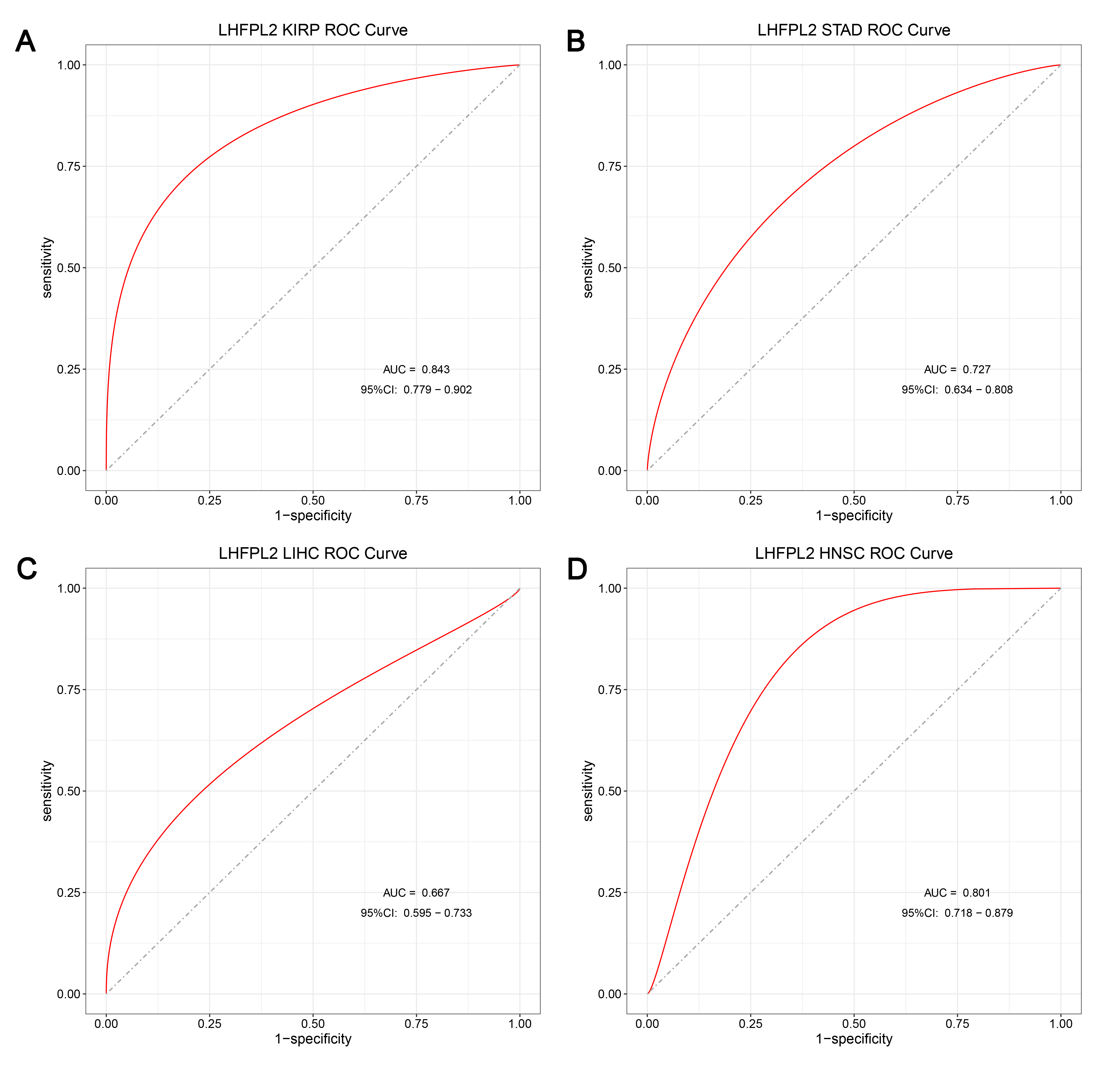

Supplement: Supplementary file 1 [file ijms-25-06707-s001.zip › ijms-3041492-supplementary/ijms-3041492-supplementary/Figure S2.tif]

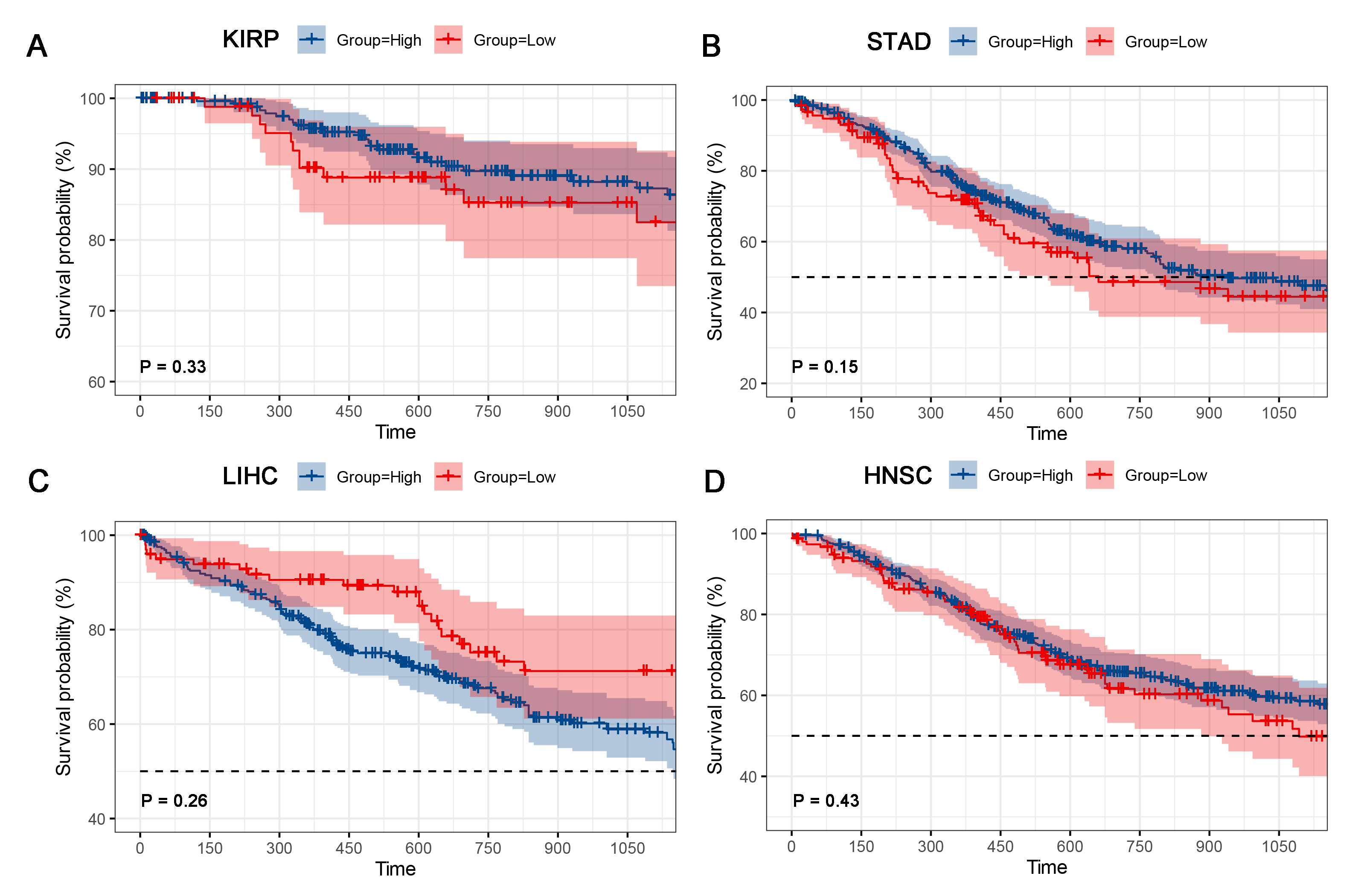

Supplement: Supplementary file 1 [file ijms-25-06707-s001.zip › ijms-3041492-supplementary/ijms-3041492-supplementary/Figure S3.tif]

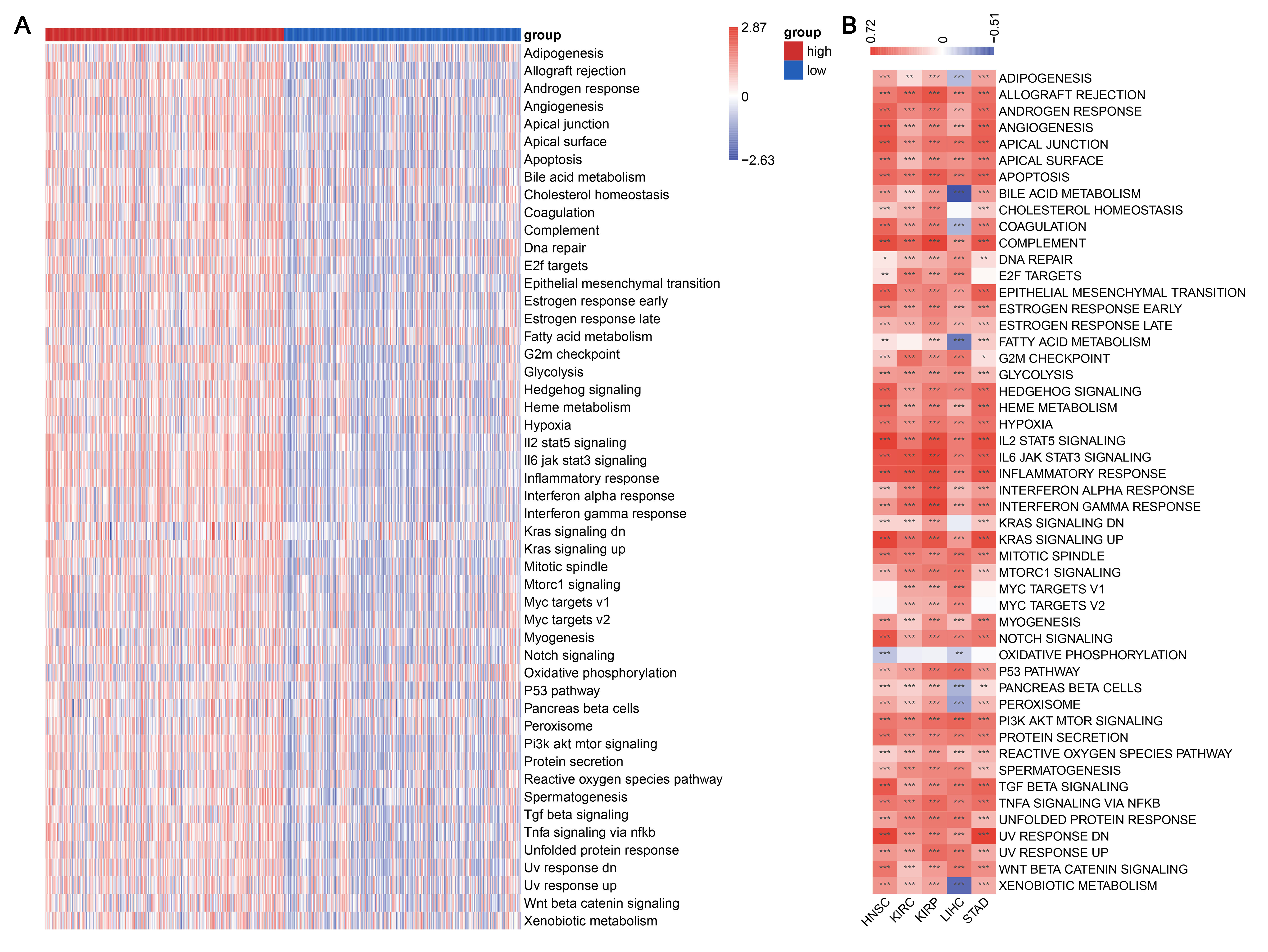

Supplement: Supplementary file 1 [file ijms-25-06707-s001.zip › ijms-3041492-supplementary/ijms-3041492-supplementary/Figure S4.tif]

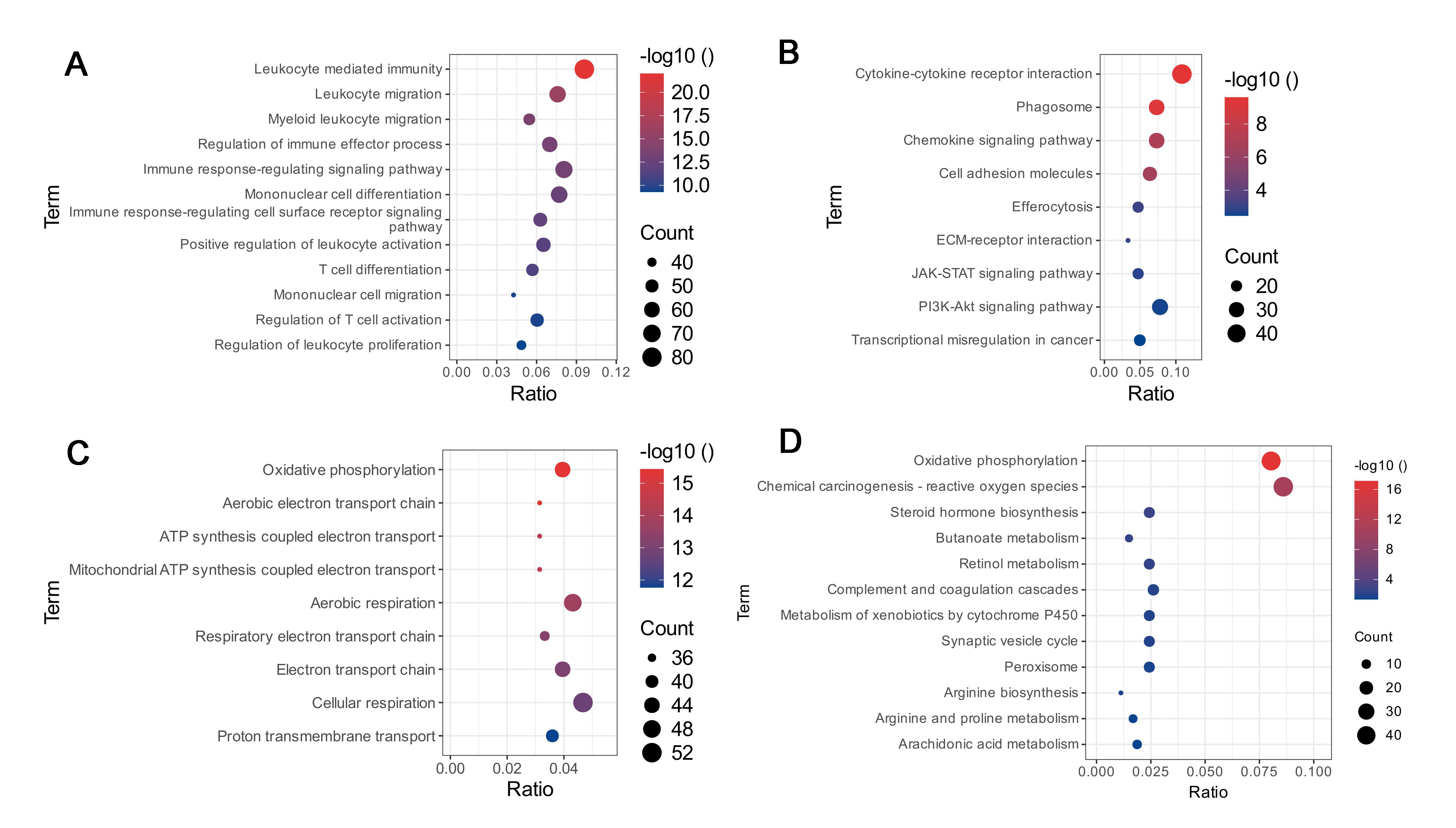

Supplement: Supplementary file 1 [file ijms-25-06707-s001.zip › ijms-3041492-supplementary/ijms-3041492-supplementary/Figure S6.tif]

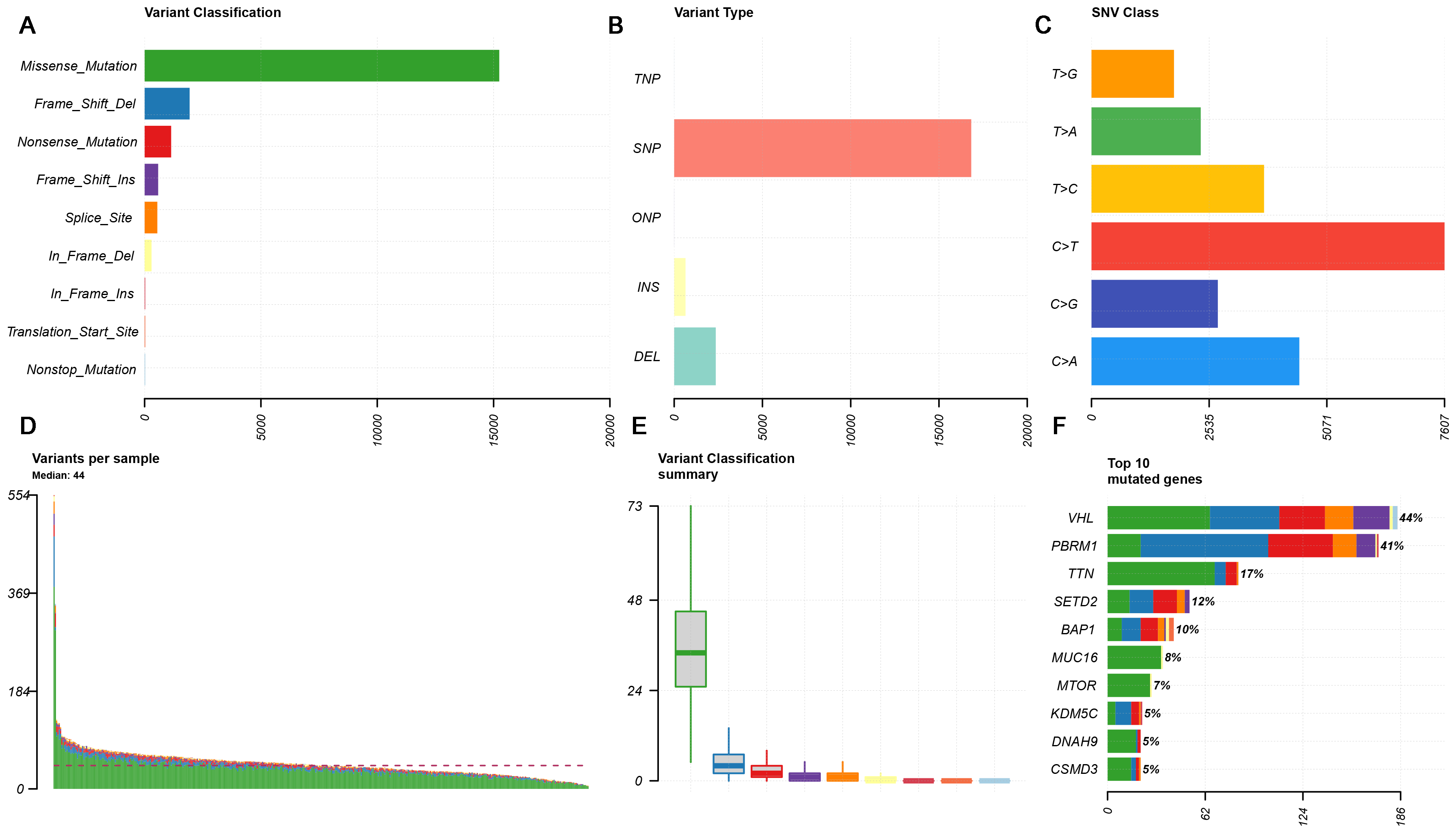

Supplement: Supplementary file 1 [file ijms-25-06707-s001.zip › ijms-3041492-supplementary/ijms-3041492-supplementary/Figure S7.tif]

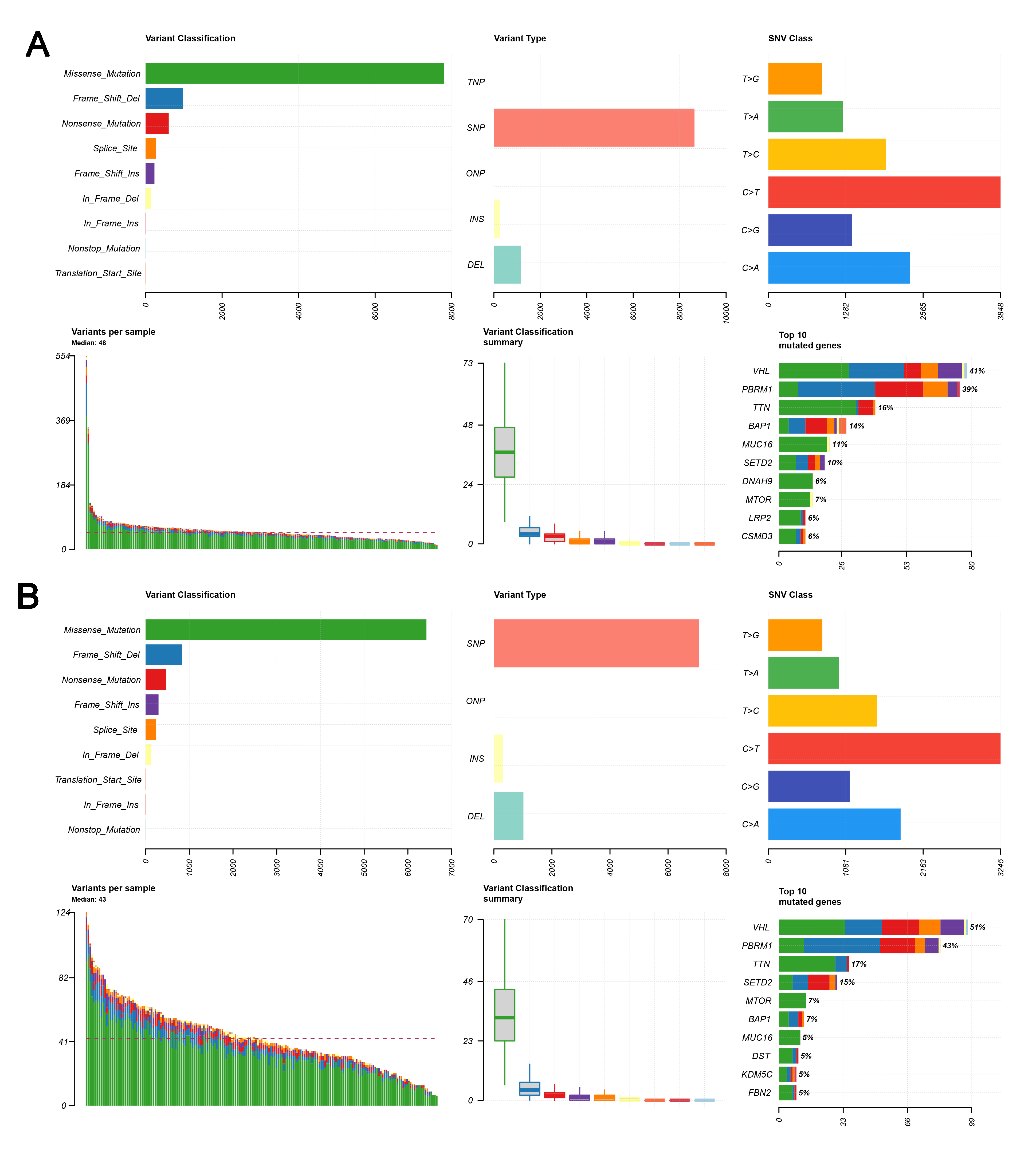

Supplement: Supplementary file 1 [file ijms-25-06707-s001.zip › ijms-3041492-supplementary/ijms-3041492-supplementary/Figure S8.tif]

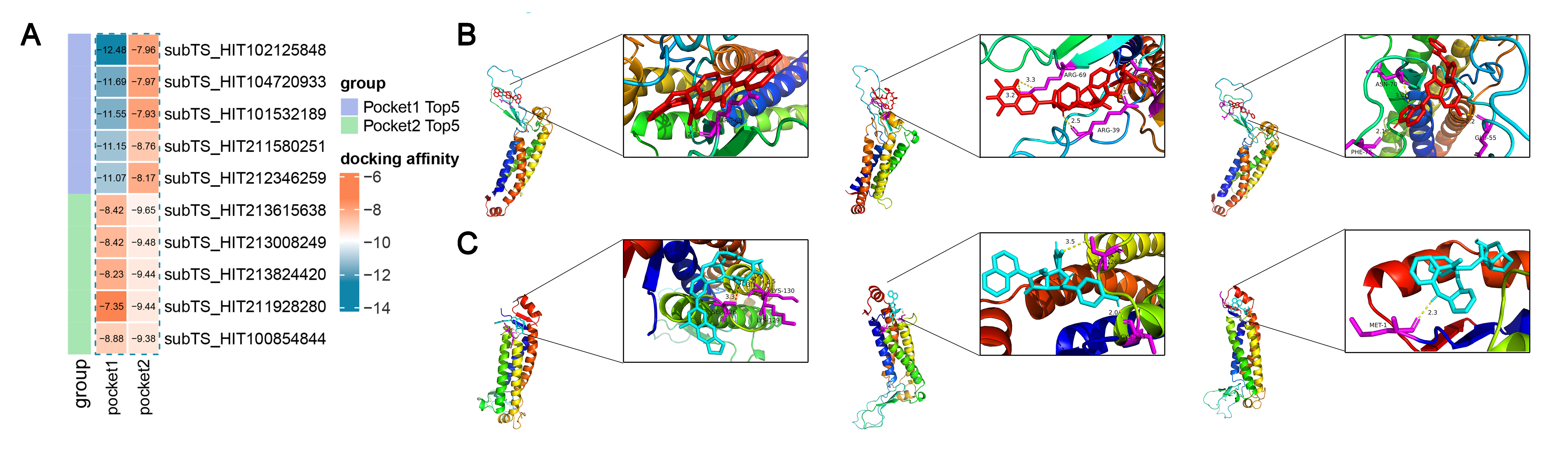

Supplement: Supplementary file 1 [file ijms-25-06707-s001.zip › ijms-3041492-supplementary/ijms-3041492-supplementary/Figure S9.tif]
